# Natural Killer (NK) Cell Functionality after human Spinal Cord Injury (SCI): protocol of a prospective, longitudinal study

**DOI:** 10.1186/s12883-016-0681-5

**Published:** 2016-09-13

**Authors:** Inês Laginha, Marcel A. Kopp, Claudia Druschel, Klaus-Dieter Schaser, Benedikt Brommer, Rick C. Hellmann, Ralf Watzlawick, Ramin-Raul Ossami-Saidi, Harald Prüss, Vieri Failli, Christian Meisel, Thomas Liebscher, Erik Prilipp, Andreas Niedeggen, Axel Ekkernkamp, Ulrike Grittner, Sophie K. Piper, Ulrich Dirnagl, Monica Killig, Chiara Romagnani, Jan M. Schwab

**Affiliations:** 1 Department of Neurology and Experimental Neurology, Charité - Universitätsmedizin Berlin, Charitéplatz 1, 10117 Berlin, Germany; 2 Clinical and Experimental Spinal Cord Injury Research (Neuroparaplegiology), Charité - Universitätsmedizin Berlin, Charitéplatz 1, 10117 Berlin, Germany; 3 Department of Musculoskeletal Surgery, Charité - Universitätsmedizin Berlin, Augustenburger Platz 1, 13353 Berlin, Germany; 4 F.M.Kirby Neurobiology Center, Childrens’s Hospital and Department of Neurology, Harvard Medical School, 300 Longwood Avenue, Boston, MA 02115 USA; 5 Institute of Medical Immunology, Charité - Universitätsmedizin Berlin, Augustenburger Platz 1, 13353 Berlin, Germany; 6 Treatment Centre for Spinal Cord Injuries, Trauma Hospital Berlin, Warener Straße 7, 12683 Berlin, Germany; 7 Deutsches Rheuma-Forschungszentrum (DRFZ), Charitéplatz 1, 10117 Berlin, Germany; 8 Department for Biostatistics and Clinical Epidemiology, Charitéplatz 1, 10117 Berlin, Germany; 9 Center for Stroke Research Berlin, Charité - Universitätsmedizin Berlin, Charitéplatz 1, 10117 Berlin, Germany; 10 Department of Neurology, Spinal Cord Injury Division, The Neuroscience Institute, The Ohio State University, Wexner Medical Center, Columbus, OH 43210 USA; 11 Department of Neuroscience and Center for Brain and Spinal Cord Repair, Department of Physical Medicine and Rehabilitation, The Neurological Institute, The Ohio State University, Wexner Medical Center, Columbus, OH 43210 USA; 12 Head Spinal Cord Injury Division, Department Neurology, The William E. Hunt and Charlotte M. Curtis Chair in Neuroscience, The Neurological Institute, The Ohio State University - Wexner Medical Center, 395 W. 12th Ave, 7th Floor, Columbus, OH 43210 USA

**Keywords:** Spinal cord injury, NK cells function, Immune paralysis, Lesion height dependency

## Abstract

**Background:**

Natural killer (NK) cells comprise the main components of lymphocyte-mediated nonspecific immunity. Through their effector function they play a crucial role combating bacterial and viral challenges. They are also thought to be key contributors to the systemic spinal cord injury-induced immune-deficiency syndrome (SCI-IDS). SCI-IDS increases susceptibility to infection and extends to the post-acute and chronic phases after SCI.

**Methods and design:**

The prospective study of NK cell function after traumatic SCI was carried out in two centers in Berlin, Germany. SCI patients and control patients with neurologically silent vertebral fracture also undergoing surgical stabilization were enrolled. Furthermore healthy controls were included to provide reference data. The NK cell function was assessed at 7 (5–9) days, 14 days (11–28) days, and 10 (8–12) weeks post-trauma. Clinical documentation included the American Spinal Injury Association (ASIA) impairment scale (AIS), neurological level of injury, infection status, concomitant injury, and medications. The primary endpoint of the study is CD107a expression by NK cells (cytotoxicity marker) 8–12 weeks following SCI. Secondary endpoints are the NK cell’s TNF-α and IFN-γ production by the NK cells 8–12 weeks following SCI.

**Discussion:**

The protocol of this study was developed to investigate the hypotheses whether i) SCI impairs NK cell function throughout the post-acute and sub-acute phases after SCI and ii) the degree of impairment relates to lesion height and severity. A deeper understanding of the SCI-IDS is crucial to enable strategies for prevention of infections, which are associated with poor neurological outcome and elevated mortality.

**Trial registration:**

DRKS00009855.

## Background

Infectious complications such as spinal cord injury (SCI) - associated pneumonia (SCI-AP) are risk factors for poor functional neurological recovery [1] and the leading cause of morbidity and mortality among SCI patients [2–4] during the post-acute and chronic phases after injury. After SCI the normally well-balanced interplay between the nervous system and immune system is disrupted resulting in a systemic Spinal Cord Injury – Induced Immunodefiency Syndrome (SCI-IDS) [5–7]. Downregulation of the immune system also occurs after other central nervous system (CNS) injuries, such as stroke and traumatic brain injury (TBI) [8, 9]. Immune depression resulting from any of the previously mentioned conditions has previously been summarised using the term CNS injury-induced immune depression syndrome (CIDS) [8–10].

Neural regulation of the immune system after SCI occurs by at least three different pathways: 1) through a neuroendocrine pathway – mainly the hypothalamic-pituitary-adrenal (HPA) axis – that coordinates the production and circulation of immunoregulatory hormones and neurotransmitters such as glucocorticoids [10–14]; 2) direct autonomic innervation through sympathetic and parasympathetic nerves of endocrine, hematopoietic and immune relevant organs such as spleen, bone marrow, and lymph nodes [12, 14–16]. 3) via cytokine synthesis at the sterile inflammatory CNS injury site and/or systemically which may elicit a systemic immune modulatory response through CNS-specific mechanisms [17, 18].

SCI-IDS was first characterized in quantitative terms [5, 6] as a reduction of leukocyte subpopulations such as T-, and B-lymphocytes and MHC class II^+^ cells, occurring during the first week after trauma as observed in both experimental models and clinical pilot SCI studies [5, 6]. However, this transient lymphopenia alone cannot be held accountable for the persistent increased susceptibility to infectious diseases in SCI patients giving rise to the possibility that a qualitative deficit might persist beyond the first week after injury.

Due to NK cells’ highly heterogenic receptor repertoire and long-lasting, specific memory [19], they belong to the first line of defence directed against a varied spectrum of intrudors including viral and bacterial antigens [19–22]. NK cells are characterized by their high cytotoxic potential, rapid activation and cytokine secretion as well as their important modulatory role over other immune competent cells, namely those related to adaptive immunity, including macrocytic “priming” and regulation of Th1/Th2 balance [22–25].

NK cells are equipped with beta-adrenergic [26] and glucocorticoid-receptors [27] that orchestrate their activity. In physiological conditions, NK cell activity is fostered after sport [28] and diminished after psychological stress in a beta-receptor-dependent manner. Corticosteroids also attenuate NK cells activity after iatrogenic surgical stress via activation of a stress/HPA axis-mediated response [29].

Subsequent to SCI deficits in NK cell function at 2 weeks, a nadir at 2 months post-injury occurs, followed by a sustained deficiency detectable up to more than a year after SCI [30, 31]. Efferent sympathetic noradrenergic/peptidergic fibers are present in the parenchyma of primary and secondary lymphoid organs including the bone marrow where they travel along vasculature ending in close proximity to precursor and hematopoietic cells [12, 13, 15]. Based on this embedded and juxtaposed neuroanatomical position it has been suggested that the autonomic nervous system (ANS) exerts an immune regulatory role. Bone marrow decentralization was shown to have an adverse effect on lymphocyte proliferation, namely on long-term colony formation [31] purportedly affecting NK cell formation and maturation in the bone marrow [21]. As only mature NK cells are able to elicit cell lysis, alterations with the maturation process of NK cells might adversely interfere with the development of their “killing machinery” and functionality.

It remains unclear whether NK cell functional impairment is caused by neurogenic-specific mechanisms. In this prospective, longitudinal multicentre study, we analyse fluctuations of NK cells in peripheral blood (quantitative aspect) together with remaining NK cell functionality (qualitative aspect). Given that NK cell function is crucial to combat viral and bacterial infections, and the ability to control infections after SCI [32] is significantly impaired, we aim to investigate whether lymphocyte mediated non-specific immunity driven by NK cells is impaired after SCI and to characterize the functional role of NK cells as a contributing factor to the SCI-IDS. We propose to measure NK cell functional parameters such as direct cytotoxicity and surrogate parameters thereof, including NK-cell activation and the capacity to mount a characteristic cytokine expression response, first, to scrutinize the degree of NK cell dysfunction induced by SCI and the one caused the post-aggression syndrome (stress and surgery) [33] and second to monitor the functional status of NK cells in the search for prognostic markers in individuals at the highest risk of developing infections. Infections have been shown to be an independent factor hindering wound healing, including the spinal cord lesion itself thereby reducing neurological recovery [1].

Driven by the lack of studies controlling for the effect of stress-/trauma- and surgery-associated mechanisms upon the NK cell function, our study aims to delineate the degree of NK cell dysfunction not directly associated with injury to the CNS by incorporating a group of patients with acute vertebral facture without SCI. Additionally, we analysed whether NK cell dysfunction is influenced by the disruption of the sympathetic outflow to the immune-relevant organs (lesion level-dependency). Furthermore, using FACS as a method, we are also able not only to measure NK cells’ cytotoxicity but additional crucial functional parameters such as cytokine production, which have not been previously assayed.

## Methods and design

### Study design, study coordination, and participating centers

This study was designed as a prospective two-center study for the detailed evaluation of NK cell activity up to 10 weeks after SCI. The coordination of this study is conducted by the Department of Experimental Neurology, Clinical and Experimental Spinal Cord Injury Research (Neuroparaplegiology) at the Campus Mitte of the Charité University Hospital, Berlin, Germany. Patients were recruited from two SCI specialized centres: the Treatment Center for Spinal Cord Injuries, Trauma Hospital Berlin, Germany and the Center for Musculoskeletal Surgery (Charité - Campus Virchow Clinic).

### Enrolment period

The recruitment and follow-up of patients started in October 2012 and was closed in December 2014. Query management, source-data verification and database clearing was performed until March 2016. Statistical evaluation and elaboration of a report are currently ongoing.

### Ethics and informed consent

The study is associated with the SCIentinel study [34] and its protocol was approved by the local Ethics Committees: Ethical Committee of the Charité – Universitätsmedizin Berlin (EA1/001/09). Participants were informed both in oral and written form about the trial and its anticipated risks and benefits, using patient information sheets. Written informed consent was obtained prior to inclusion in the study. This study complies with the Helsinki Declaration in its recent German version, the Medical Association code of conduct, the principles of Good Clinical Practice (GCP) and the Federal Data Protection Act. The study is carried out according to local legal and regulatory requirements. The trial protocol was registered in the German clinical trials registry (DRKS00009855).

### Participants

**Fig. 1:**
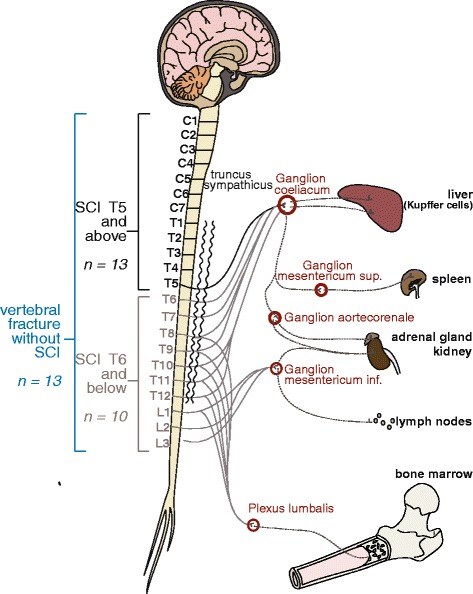
Numbers of study participants, groups and innervation of immune relevant organs. Recruited patients were allocated to 3 different cohorts: i) patients with a SCI lesion at level Th5 and above (*black*); ii) patients with a SCI lesion at level Th6 and below (*grey*); iii) neurologically silent patients with a vertebral fracture at any level. SCI-lesions at Th5 and above (i), depending on the completeness of the lesion (AIS A-D), result in a partial or total denervation of the sympathetic pregranglionic neurons synapsing on the celiac ganglion and subsequent ganglia connected to the segments below the lesion. In patients with lesions at Th6 and below (ii), the innervation of the celiac ganglion remains completely/partially intact. In the third group the sympathetic innervation of neuroendocrine or primary and secondary immune organs remains intact (iii)

A total of four groups were enrolled in the study. In order to investigate whether the extent of NK cell dysfunction is lesion level dependent, SCI patients were separated in 2 groups i) patients with rostral lesions (Th5 or above) and ii) patients with caudal lesions (Th6 or below), with a different degree of immune relevant organ denervation and sympathetic outflow impairment. Alterations in NK cell activity have been described not only in cases of CNS disturbances [9, 13, 35] but also after surgical procedures [29]. Therefore a control group iii) including patients with an acute vertebral fracture, undergoing stabilization surgery without SCI was enrolled in order to distinguish neurogenic immune suppression from stress (post-aggression-syndrome) associated altered NK cell activity (Fig. 1). Reference values for the NK cell function were given by an additional group (iv) of healthy controls.

The estimated frequency of patients initially treated with methylprednisolone in the recruiting centres is expected to be between 5 and 10 %. These patients will be excluded from the analysis of the primary endpoint given the well-known suppressive effect of glucocorticoids on NK cells function [29].

### Sample size calculation

The number of patients to be enrolled in this study was calculated based on a previous study of Iversen and colleagues (*n* = 18) analysing growth and activity of leucocytes following SCI [31]. With regard to the comparison between 12 patients and 6 controls Iversen et al. reported an effect size of around 1.6 (Cohen’s d) in lymphocyte cytoxiticity (patients mean ± SEM,: 5.2 ± 0.7 and 4.5 ± 0.7 g/L in the paraplegics and tetraplegics patients with *n* = 6, respectively, versus 8.2 ± 0.8 g/L in the control subjects, *n* = 6).

The primary endpoint in this study is the difference of NK cell cytotoxicity (NK cell expression of CD107a) during the sub-acute phase (assessed between 8 and 12 weeks after SCI) between all SCI patients and controls. The sample size calculation (software R package ‘pwr’, https://cran.r-project.org/web/packages/pwr/pwr.pdf) was performed assuming an effect size of 1.0 for the difference between all SCI patients (either with rostral lesions Th5 or above, (group i) or with caudal lesions Th6 or below (group ii) versus control patients with acute vertebral fracture, but without SCI (group iii). Type I error was set to 0.05 (two-sided); type II error to 0.2 (80 % Study Power). To test the main hypothesis with a two sided *t*-test, a total sample size of 36 SCI patients (pooled group i and ii, *n* = 23) and controls (group iii, *n* = 13) would be required. Aiming for adequate power for the testing of secondary hypotheses between the two SCI patient groups with different lesion levels and to account for possible drop out we aimed to include at least 15–20 patients in each of the three groups. Furthermore, we included a small group of healthy controls (*n* = 10) as a reference group (group iv).

### Enrolment and eligibility criteria

After admission to the hospital, the patients were subject to a clinical examination and an interview to establish eligibility for the study. As this is a study associated with the SCIentinel study [34] the selection criteria were predefined accordingly and are in line with those of previously published studies [6, 36]. Study enrolment is in accordance with the following eligibility criteria: Patients admitted to this study must be 18 years or older with acute isolated SCI after decompression or stabilization surgery, or with a neurologically silent vertebral fracture after stabilization surgery. Eligibility criteria were established in order to minimize bias and to obtain representative data. Patients with extensive polytrauma or TBI are to be excluded as these lesions may also have a detrimental effect on the immune system. Exclusion of patients with conditions that chronically induce changes on the immune system such as clinical history of neoplasia, pre-existing infections, autoimmune disease or chronic intake of corticosteroids limits the number of possible confounding factors.

### Documentation schedule

Study enrolment is documented in Case Report Forms (CRFs). These include information about neurological classification, injury date and time, medical history, concomitant injury, medication history, acute SCI therapy concerning high-dose methylprednisolone treatment as well as details of surgical intervention. Blood sampling took place at 3 different time slots after the lesion as described in the section “observational period” and was scheduled between 7:00 and 11:00 a.m. to minimize the influence of circadian rhythm on the tested parameters.

### Neurological classification

Neurological evaluation was performed according to the International Standards for Neurological Classification of Spinal Cord Injury Patients (ISNCSCI) [37], a revision of the American Spinal Injury Association (ASIA) classification in order to standardize the characterization of the lesion by the collaborating centres. ISNCSCI comprises the assessment of completeness of lesion – ASIA Impairment scale (AIS) – and the single neurological level of the lesion.

### Definition of infections

Another important clinical measure with crucial importance for this study is the diagnosis of infectious diseases. SCI-associated pneumonia and urinary tract infections (UTIs) are the most prevalent infections within the SCI population and are diagnosed and documented according to published definition of disease to ensure comparability [38, 39]. UTI is defined as bacteriuria greater than 10^5^ cells/ml or WBC count of more than 100/mm^3^ - according to the National group on urologic rehabilitation of paraplegics [38]. Pneumonia was diagnosed on the basis of the finding of opacities and infiltrates in chest X-ray.

### Target cell population

K562 is a human leukemic cell line devoid of MHC class I and are therefore specific targets of NK cells [40]. They are obtained from Deutsches Rheuma-Forschungszentrum (DRFZ; courtesy of the Romagnani group) and kept under sterile conditions in the Dept. of Experimental Neurology. K562 are cultured in RPMI-1640 (Gibco) supplemented with 10 % fetal calf serum (FCS) (lonza) and Penicillin (100U/ml, Biochrom) Streptavidin (100 μg/ml, Biochrom). The cell cultures are maintained at 37 °C, 5 % CO_2_.

### Blood sample handling

Peripheral blood was drawn under sterile conditions from each participant at the specified time points. All samples are anonymized with a six-figure pseudonym and any personal information of the participants is removed. One 8 ml BD Vacutainer® CPT™/Ficoll™ tube was collected from each admitted patient who fulfils the inclusion criteria for immediate preparation of peripheral blood mononuclear cells (PBMCs).

### NK cell stimulation assays [23–25, 40]

Before stimulation, the frequency of NK cells was calculated by flow cytometry (FACScalibur, BD biosciences) and the effector:target cell ratio was established. In a sterile environment, four different conditions were prepared each in the concentration of 10.000 PBMCs/μl in FACS tubes: i) with K562 cells in 5:1 effector:target cell ratio; ii) with phorbol-12-myristate-13-acetate (PMA) (20 ng/ml) (Sigma Aldrich) and ionomycin (1 μg/ml) (Sigma Aldrich); iii) with interleukin (IL)-12 (50 μg/ml) and IL-18 (50 μg/ml); and iv) unstimulated control with medium without additional additives. To all four conditions, CD107a FITC (2 μg/ml) (BD Pharmingen, BD Biosciences) and monensin (0,7 μg/ml) (BD GolgiStop, BD Biosciences) were added. Cells were then incubated at 37 °C in 5 % CO_2_. After 1 h of incubation Brefeldin A (BFA) (10 μg/ml) (Sigma Aldrich) was added to each tube and incubation continued for yet another 5 h at 37 °C in 0,5 % CO_2_. Brefeldin A was added to prevent spontaneous exocytosis of cytokine-containing vesicles to enable intracellular staining, whilst monensin was added to prevent the acidification of the cytokines inside the vesicles and any subsequent degradation of CD107a when internalized. After 6 h of stimulation, PBMCs were labelled for NK cell marker expression CD3 PerCP cy5.5 (Biolegend), CD56 APC (BD biosciences) and a fixable yellow dead cell marker (Invitrogen) for 20 min. Cells were then fixed and permeabilized according to manufacturers instructions (BD Biosciences) and stained for IFN-γ PE cy7 (BD Pharmingen, BD Biosciences) and TNF-α efluor 450 (ebioscience) for 30 min. Cells were then resuspended in PBS/BSA and measured using fluorescence-activated cell sorting (FACS) Canto (BD Bisciences) in the FACS facility of the DRFZ and acquired with DivaSoftware. A total of 20,000 to 50,000 NK cells per tube were recorded. Analysis was made using FlowJo software version 8.7 for Mac. The gating was performed as follows: gating on lymphocyte population, exclusion of dead cells, and selection of CD3^−^ CD56^+^ cells. After adequately compensated, the expression of CD107a^+^, IFN-γ^+^, TNF-α^+^ was individually measured [40].

### Observation period

**Fig. 2:**
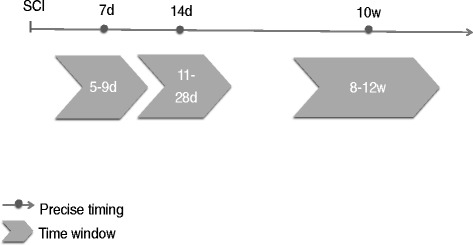
Scheduled patient’s visits. Blood collection, patient documentation and NK cell assays was performed during indicated time-windows. The timeline is starting at the time of injury (SCI)

The first observational time point was chosen with respect to the expected time when the quantitative immune depression recedes according to the SCI-IDS pilot study [6]. Three time windows were elected for blood sampling and subsequent analysis to provide insight about late acute, postacute and sub-acute phases post-trauma: day 5 to 9 (mentioned as week 1) day 11 to 28 (mentioned as week 2) and 8th to 12th week (mentioned as week 10) after the injury (Fig. 2). These were selected because they corresponded to the hallmarks of parenchymal inflammation following SCI and were in line with previous trials where immunological changes had also been observed [36].

### Primary and secondary endpoints

The primary endpoint is to analyse NK cytotoxicity during the sub-acute phase (8–12 weeks) after SCI. NK cells cytotoxic capacity was measured by quantifying the expression of CD107a, a molecule expressed by exocytosis of the cytotoxic granules.

NK cell 107a expression can be elicited by two ways: 1) Exposure of NK cells to specific target cells – K562, an erythroleukemic cell line devoid of MHC I [24, 25, 40, 41]; 2) PMA and ionomycin stimulation [40]. PMA is a robust lymphocyte activation stimulus bypassing membrane receptors acting through protein kinase C activation pathway and ionomycin is a calcium ionophore [24, 40].

Secondary endpoints of the study are the quantification of the NK cell’s IFN-γ and TNF-α production during the subacute phase. IFN-γ and TNF-α production can be triggered through a well-established immunological challenge: PMA and ionomycin stimulation [22]. The released IFN-γ and TNF-α synthesis denotes the intrinsic potential of each NK cell to produce cytokines. The frequency of degranulating and cytokine-producing NK cells was measured by multicolour flow cytometry.

### Data and statistical analysis

Administrative intermediate feasibility evaluations were performed during the enrolment period to assure appropriateness of the recruitment schedule.

Data analysis of the primary endpoint consists of a two group comparison between all SCI-patients (groups i and ii) and the control group of patients with acute vertebral fracture (group iii). Furthermore, in secondary analyses we plan the comparison between the 3 separate patient groups (i-iii) and the reference group of healthy controls (group iv) over the 3 consecutive time intervals using adjusted linear mixed models as implemented in the software package SPSS (IBM Corp. Released 2013. IBM SPSS Statistics for Windows, Version 22.0. Armonk, NY: IBM Corp.). The analysis was performed using the full dataset comprising all patients included according to the criteria as defined in the study protocol.

### Outcomes

The aim is to identify functional NK cells impairment as a component of lymphocyte-mediated non-specific immunity at different stages after injury with a more in depth consideration from different stages after SCI. The purpose is to better characterize SCI-IDS, establish potential prognostic criteria in patients at highest risk for SCI-associated infections, and possibly suggest novel preventive therapeutic approaches for SCI patients, including vaccination.

Furthermore, in order to challenge the hypothesis of a neurogenic origin of NK dysfunction we also aim to establish a correlation between the lesion level and severity with the extent of NK dysfunction. Ultimately the results from this study might influence guidelines regarding early recognition of the SCI-IDS and diagnosis of subsequent infections leading to targeted immune and antibiotic therapies in order to enhance functional neurological recovery in SCI patients.

## Discussion

This prospective study investigates the influence of SCI on NK cell activity taking into account its progression through established disease phases as well as a dependency on lesion level and the sympathetic innervation of immune relevant organs. The aim is to characterize SCI-IDS in qualitative terms beyond the first week following injury, investigating not only cell counts but also cellular function [5–7]. We hypothesize that disruption of spinal cord sympathetic outflow causes a pronounced protracted immune deficiency by targeting NK cell function and extending throughout the acute, post-acute and sub-acute phases following SCI. Furthermore, we hypothesize that patients with a Th5 lesion or higher, located above the sympathetic outlet of the immune relevant organs conveyed by the celiac and subsequent paravertebral ganglia, have a lower residual NK cells function than patients with a lesion at Th6 or lower, where the sympathetic preganglionic neurons are partially or completely intact. Moreover, we hypothesize that NK cells of patients with a vertebral fracture (controls) without any neurological deficit perform better than those in the other two groups. In contrast to previous studies and by using FACS, we address the question whether additional NK cell functions beyond cytotoxicity, such as cytokine production were compromised by SCI during the study period.

## Abbreviations

AIS: American Spinal Injury Association Impaierment Scale
ANS: autonomic nervous system
ASIA: American Spinal Injury Association
BCT: blood collection tube
CRF: case report form
DRFZ: Deutsches Rheuma-Forschungszentrum
EDTA: ethylenediaminetetraacetic acid
GCP: good clinical practice
HPA: hypothalamic-pituitary-adrenal axis
IFN-γ: interferon-gamma
LAMP-1: lysosome-associated membrane protein-1
NK: natural killer
PMA: phorbol-12-myristate-13-acetate
RPMI: Roswell Park Memorial Institute
SCI: spinal cord injury
SCI-AP: spinal cord injury- associated pneumonia
SCI-IDS: spinal cord injury – induced immune depression syndrome
SNS: sympathetic nervous system
TBI: traumatic brain injury
TNF-α: tumor necrosis factor-alpha
UTI: urinary tract infection
